# Helicobacteraceae in Bulk Tank Milk of Dairy Herds from Northern Italy

**DOI:** 10.1155/2015/639521

**Published:** 2015-05-18

**Authors:** Valentina Bianchini, Camilla Recordati, Laura Borella, Valentina Gualdi, Eugenio Scanziani, Elisa Selvatico, Mario Luini

**Affiliations:** ^1^Istituto Zooprofilattico Sperimentale della Lombardia e dell'Emilia Romagna, 26900 Lodi, Italy; ^2^Mouse and Animal Pathology Laboratory, Filarete Foundation, 20139 Milan, Italy; ^3^Genomics Platform, Parco Tecnologico Padano, 26900 Lodi, Italy; ^4^Department of Veterinary Science and Public Health, University of Milan, 20133 Milano, Italy

## Abstract

*Helicobacter pylori* is responsible for gastritis and gastric adenocarcinoma in humans, but the routes of transmission of this bacterium have not been clearly defined. Few studies led to supposing that *H. pylori* could be transmitted through raw milk, and no one investigated the presence of other Helicobacteraceae in milk. In the current work, the presence of Helicobacteraceae was investigated in the bulk tank milk of dairy cattle herds located in northern Italy both by direct plating onto *H. pylori* selective medium and by screening PCR for Helicobacteraceae, followed by specific PCRs for *H. pylori*, *Wolinella* spp., and “*Candidatus Helicobacter bovis*.” Three out of 163 bulk milk samples tested positive for Helicobacteraceae, but not for the subsequent PCRs. *H. pylori* was not isolated in any case. However, given similar growth conditions, *Arcobacter butzleri*, *A. cryaerophilus*, and *A. skirrowii* were recovered. In conclusion, the prevalence of Helicobacteraceae in raw milk was negligible (1.8%), and *H. pylori* was not identified in any of the positive samples, suggesting that, at least in the farming conditions of the investigated area, bovine milk does not represent a potential source of infection.

## 1. Introduction

*Helicobacter pylori* colonizes the stomach of approximately one half of the world population and is involved in the pathogenesis of several diseases, such as chronic gastritis, peptic ulcers, gastric adenocarcinoma, and mucosa-associated lymphoid tissue tumours [1]. The routes by which* H. pylori* is transmitted have not been firmly established, but different studies support the direct oral-oral transfer or the indirect faecal-oral transmission [2, 3]. Recently, some studies suggested that* H. pylori* can colonize the gastrointestinal tract of domestic ruminants, which could act as reservoirs and transmit the microorganism through contaminated milk. This was firstly proposed by Dore and colleagues [4], who detected the DNA of* H. pylori* in 60.3% of individual sheep milk and were able to culture the bacterium from one sample. Similarly, in Japan, Fujimura et al. [5] demonstrated the presence of* H. pylori* DNA in 72% of raw and 55% of pasteurized bovine milk and isolated one strain. Later on, in Southern Italy, Quaglia and coworkers reported a prevalence of* H. pylori* of 50%, 25.6%, and 33% in bovine, ovine, and caprine bulk milk [6] and of 6% in sheep gastric mucosa [7], but no isolates were obtained after culture of the PCR-positive samples. Also Angelidis et al. [8] detected* H. pylori* in 20% of bovine bulk tank milk by fluorescence* in situ* hybridization, while Safaei et al. [9] reported a prevalence of 16 and 40%, respectively, in individual milk and bovine feces based on PCR and antigen detection tests. On the contrary, both Jiang and Doyle [10], and Turutoglu and Mudul [11] failed to detect* H. pylori* in bovine and ovine raw milk in the US and in Turkey, respectively, by PCR and bacteriological analysis, so the hypothesis that* H. pylori* is transmitted with contaminated milk is still debated.

With the exception of* H. pylori*, no investigation on the presence of other Helicobacteraceae in the milk has been so far performed and very few studies researched Helicobacteraceae in gastrointestinal tract of cattle.* Wolinella succinogenes*, which belongs to the family Helicobacteraceae, was originally isolated from cattle rumen [12], and “*Candidatus Helicobacter bovis*” was described in the pyloric portion of the abomasum [13]. The study of Helicobacteraceae other than* H. pylori* in animals is valuable because some* Helicobacter* spp. (e.g.,* H. heilmannii*,* H. suis*,* H. felis*, and* H. pullorum*) have a zoonotic potential and are responsible for gastrointestinal disorders, and rarely bacteremia in humans [14].

The aim of this study was to investigate the prevalence of* H. pylori* and other Helicobacteraceae in raw milk of dairy cattle of an intensive farming area in order to assess the potential zoonotic role of milk in the transmission to humans of these bacteria, especially because in this area the consumption of raw milk purchased by self-service automatic vending machines represents a common practice.

## 2. Material and Methods

### 2.1. Sampling

Milk samples were collected between September and December 2013 from the bulk milk tanks of 163 dairy herds of Lodi Province (located in northern Italy) undergoing the routine monitoring programs. In this area, the average herd size was 150 milking cows and annual milk production per animal 9,000 kg. The milk samples were transported chilled to the laboratory and processed within 6 hours after the collection.

### 2.2. Microbiological Analysis

A 50 *μ*L aliquot of the samples was streaked onto* H. pylori* selective medium, containing Columbia Blood Agar base, 7% laked horse blood, and DENT Supplement (all from Oxoid, Ltd., Basingstoke, United Kingdom), prepared according to the manufacturer's instructions. The plates were incubated for seven days at 37°C in a microaerophilic atmosphere (GENbox; bioMérieux, Marcy l'Etoile, France). Colonies with typical morphology (small or very small, round, or translucent colonies) were subcultured and subjected to Gram staining. Gram-negative spiral-shaped rods were subjected to species identification by molecular analysis.

### 2.3. Molecular Analysis

DNA was extracted from milk samples slightly modifying the method reported by Graber and colleagues [15]. This protocol should ensure both the release of intracellular or cellular-adhered bacteria and the detachment of bacteria stuck to the fat globules. Briefly, 625 *μ*L of Triton X-100, 312.5 *μ*L of 1% trypsin solution, and 375 *μ*L of* Lactobacillus casei* (4·10^10^ CFU) were added to 1 mL of each milk sample. The specimens were incubated at 55°C for 15 min and centrifuged for 15 min at 4000 g. Afterwards, the supernatant was discarded, the pellet was washed with 1 mL of 1x phosphate buffered saline, and the DNA was extracted using the PureLink Genomic DNA Mini Kit (Life Technologies, Paisley, United Kingdom).

The 16S rRNA gene of members of the family Helicobacteraceae was amplified by PCR using primers C97-C98 [16], followed by a nested PCR using the internal pair of primers HelF-HelR2 [17]. Samples positive for Helicobacteraceae were confirmed as positive with primers O68 and M86 [18] targeting the 23S rRNA* Helicobacter *gene and further tested for the presence of* H. pylori*,* Wolinella *spp., and “*Candidatus Helicobacter bovis*” using the PCR method described by Quaglia et al. [6], Craven et al. [19], and De Groote et al. [13], respectively.

*H. pylori* ATCC 43504 and* W. succinogenes* LMG 7466 were used as positive controls in the respective specific PCRs and in the PCR for Helicobacteraceae. Due to uncultivability of “*Candidatus Helicobacter bovis*” a reference strain is not available; thus the specific PCR was applied to the DNA isolated from the abomasum of an infected cow [20], and PCR products were sequenced with the same primers used for amplification. Comparison of the sequence to the NCBI database through the algorithm BLAST (http://www.ncbi.nlm.nih.gov/BLAST) revealed a 99% similarity with partial sequence of 16S rRNA gene of “*Candidatus Helicobacter bovis*” (accession number: AF317470.1, AF127027.1). This DNA served as positive control in the subsequent PCR reactions and in the PCR for Helicobacteraceae.

Species identification of the cultured strains was performed by 16S rRNA gene sequencing. Sequencing reaction setup was performed in a final volume of 10 *μ*L with Big Dye Terminator kit v 3.1 Chemistry (Applied Biosystems, Paisley, United Kingdom) following protocol instruction. Capillary electrophoresis was performed on ABI 3730 DNA Analyzers (Applied Biosystems). DNA sequences were analyzed with the software BioEdit (http://www.mbio.ncsu.edu/BioEdit/bioedit.html); for each positive PCR, consensus sequences were obtained with the algorithm CAP (Contig Assembly Program) [21]; the identity of the 16S rRNA gene sequences was verified by comparison of the consensus sequences to the NCBI database through the algorithm BLAST.

## 3. Results and Discussion

An overview of the results is shown in Table 1. Three out of 163 bulk tank milk samples tested positive by PCR for Helicobacteraceae (1.8%, CI 95%: 0–3.9%). However these three samples reacted negatively for any of the subsequent species-specific PCRs and no* Helicobacter *spp. were cultured. In four Helicobacteraceae PCR-negative cases a variable number (10–100) of colonies with morphology referable to* Helicobacter *were cultured. Gram staining showed spiral-shaped rods, but species identification revealed that the bacterial isolates were* Arcobacter butzleri* (*n* = 2),* Arcobacter cryaerophilus *(*n* = 1), and* Arcobacter skirrowii *(*n* = 1).

*H. pylori* is involved in several human diseases, but the routes by which the pathogen is transmitted have not been fully determined, although oral-oral and fecal-oral transmission are usually suspected [3]. By molecular analysis, some surveys reported a high prevalence of* H. pylori* in raw or pasteurized bovine milk [6, 8], but only very few cases of successful isolation of the strain were described [4, 5]. Conversely, other researches failed to detect the pathogen in milk [10, 11]. In the current study, 163 bovine bulk milk samples were assayed for the presence of* H. pylori* both by direct plating onto selective medium and by PCR, but the bacterium was not detected in any of the samples. The wide difference between the reported prevalences might be due to the different methods of analysis. We cultured a low volume of milk in order to restrict the overgrowth of contaminating microflora. Unlike a previous study [22], an enrichment process was not applied, because this medium contains nalidixic acid, to which different* Helicobacter* spp. were susceptible [23], and we were interested in culturing not only* H. pylori*, but also other* Helicobacter* species. Moreover, since our samples were transferred chilled to the laboratory, a conversion of* H. pylori* cells to viable but nonculturable (VBNC) forms might have occurred and should be considered responsible for the failed isolation of* H. pylori* from the examined samples, even though different studies demonstrated that* H. pylori* may survive for some days in milk under refrigeration [24–26]. More likely, the difference in reported prevalences can be related to the different geographic areas or farming conditions (i.e., animal-human promiscuity, contemporary housing of cows and small ruminants, and hygienic standards) where the investigations were performed. Moreover, due to the high prevalence of* H. pylori*-infected people, it cannot be excluded that milk contamination occurred during the milking process through carrier milkers.

In the present study three samples tested positive for Helicobacteraceae PCR. As “*Candidatus Helicobacter bovis*” is present at high levels in cattle abomasa [13], one might expect a transit into the intestinal tract and fecal excretion. A similar situation is expected for* Wolinella *spp., which are found in cattle rumen [12]. Personal observations confirm that such bacteria can be frequently found in the gastrointestinal tract of cattle of our region [20]. Since the presence of microorganisms in bulk milk seems to be mainly associated with fecal contamination [27], we evaluated the presence of these two bacteria species in the three milk samples positive for Helicobacteraceae. “*Candidatus Helicobacter bovis*” and* Wolinella *spp. were not detected in any of the samples, suggesting that other Helicobacteraceae can be found in the milk.

No* Helicobacter* spp. were found by bacteriological analysis. This could be due to the overgrowth of contaminating microflora, which prevents the identification of the small colonies referable to* H. pylori*, or to the presence of VBNC forms. Another reason could be the higher sensitivity of the molecular analysis with respect to culture method, which allows also the detection of low number of* Helicobacter* cells in milk samples. Furthermore, some* Helicobacter *species, including “*Candidatus Helicobacter bovis*,” are yet uncultivable in common culture media. However, given similar growth conditions,* Arcobacter* spp. were isolated in four samples. This is noteworthy because* Arcobacter* spp. (which are closely related to* Campylobacter* and* Helicobacter* genera) are considered emergent enteropathogens and potential zoonotic agents [28].

Even though the specificity of the PCRs for Helicobacteraceae (primers C97-C98, and Hel3-Hel4 for the nested PCR) was confirmed* in silico*, one out of the four isolated* Arcobacter* strains (*A. butzleri* IZSLER-260361) cross-reacted with primers C97-C98, while the milk from which it was isolated was negative. Also the 23S rRNA* Helicobacter* gene PCR [18] amplified all the four* Arcobacter* strains isolated in this study, as well as* Campylobacter jejuni* ATCC 49943 (data not shown). According to these findings, these primers targeting 16S and 23S rRNA genes of* Helicobacter *spp. can react unspecifically with non-Helicobacteraceae species. Thus the detection of Helicobacteraceae from the three bulk tank milk samples of our study should be considered questionable, raising concerns also about the results obtained from milk with the cited 16S rRNA gene targeting PCR reported in the literature [29]. Further studies based on a metagenomic approach will be able to confirm or exclude the presence of Helicobacteraceae in these positive bulk tank milk samples, providing also an identification at species level.

## 4. Conclusions

The results of this study revealed that* H. pylori* is not present in the bulk tank milk and indicated a negligible prevalence of Helicobacteraceae in raw milk of dairy cattle, suggesting that milk is not a transmission vehicle of this infection, at least in the examined geographic area and under the investigated farming conditions.

## Figures and Tables

**Table 1:** Results of molecular and bacteriological analysis performed on 163 bulk tank milk samples.

Number of milk samples	PCR					Bacteriological analysis^b^
	Helicobacteraceae		*H. pylori* ^a^	“*Candidatus Helicobacter bovis*”^a^	*Wolinella* spp.^a^
	16S rRNA	23S rRNA^a^			
156	−	nd	nd	nd	nd	−
3	+	+	−	−	−	−
2	−	+	nd	nd	nd	*Arcobacter butzleri *
1	−	+	nd	nd	nd	*Arcobacter cryaerophilus *
1	−	+	nd	nd	nd	*Arcobacter skirrowii *

^a^PCRs for 23S rRNA gene of *Helicobacter*, *H. pylori*, “*Candidatus Helicobacter bovis,*” and *Wolinella* spp. were performed on samples positive for the screening PCR for 16S rRNA gene of Helicobacteraceae. ^b^Strain identification was performed by 16S rRNA gene sequencing (nd: not done).
